# Exploration of inactive metabolic pathways in Antarctic *Pseudogymnoascus australis* through elicitation: a genomic and metabolomic approach to investigate its biotechnological potential

**DOI:** 10.3897/imafungus.17.156018

**Published:** 2026-02-11

**Authors:** Karla Leal, Juan Machuca, David Madariaga, María José Contreras, Leticia Barrientos, Kattia Nuñez-Montero, Estefanía Chavarría, Pablo Bruna, Isabel Iturrieta-González

**Affiliations:** 1 Instituto de Ciencias Aplicadas, Facultad de Ingeniería, Universidad Autónoma de Chile, Temuco 4810101, Chile Facultad de Ingeniería, Universidad Autónoma de Chile Temuco Chile; 2 Doctorado en Ciencias Biomédicas, Facultad de Ciencias de la Salud, Universidad Autónoma de Chile, Temuco 4810101, Chile Facultad de Ciencias de la Salud, Universidad Autónoma de Chile Temuco Chile; 3 Instituto de Ciencias Aplicadas, Facultad de Ciencias de la Salud, Universidad Autónoma de Chile, Temuco 4810101, Chile Instituto de Ciencias Aplicadas, Facultad de Ciencias de la Salud, Universidad Autónoma de Chile Temuco Chile; 4 Centro de Investigación en Biotecnología, Escuela de Biología, Instituto Tecnológico de Costa Rica, Cartago, Costa Rica Escuela de Biología, Instituto Tecnológico de Costa Rica Cartago Costa Rica; 5 Programa de Doctorado en Ciencias Mención Biología Celular y Molecular Aplicada, Universidad de La Frontera, Temuco 4811230, Chile Programa de Doctorado en Ciencias Mención Biología Celular y Molecular Aplicada, Universidad de La Frontera Temuco Chile; 6 Departamento de Ciencias Preclínicas, Facultad de Medicina, Laboratorio de Infectología e Inmunología Clínica, Centro de Excelencia en Medicina Traslacional-Núcleo Científico y Tecnológico (CEMT-BIOREN), Universidad de La Frontera, Temuco 4810296, Chile Centro de Excelencia en Medicina Traslacional-Núcleo Científico y Tecnológico (CEMT-BIOREN), Universidad de La Frontera Temuco Chile

**Keywords:** Biosynthetic gene clusters, *Pseudogymnoascus australis*, psychrophilic fungi, secondary metabolites

## Abstract

This study presents the first annotated genome of the Antarctic fungus *Pseudogymnoascus australis* UA-032-E, revealing significant biosynthetic potential with 44 predicted biosynthetic gene clusters (BGCs) identified through antiSMASH analysis. These BGCs include nonribosomal peptide synthetases (NRPS), type I polyketide synthases (PKS), and hybrid systems, indicating a diverse capacity for secondary metabolite production. BiG-SCAPE analysis grouped these clusters into 41 gene cluster families, with most being singletons, demonstrating low genetic redundancy and high structural diversity. To activate silent pathways, we employed elicitors (NPS and LPS) across multiple culture media, successfully inducing previously undetected metabolic activity. Using an integrated LC–QTOF–MS/MS approach combined with the GNPS and SIRIUS platforms, a total of 75 features were detected, including cyclodipeptides [cyclo-(Pro-Val), cyclo-(Leu-Leu)], maculosin, and betaine lipids such as DGTS 18:2—compounds linked to stress adaptation and biological activities in the literature. The YES medium supplemented with LPS elicitation yielded the highest metabolic diversity, suggesting this combination effectively stimulates specialized metabolism. Our findings demonstrate the value of combining genomic and metabolomic approaches to unlock the chemical potential of psychrophilic fungi. The genomic resource presented here provides a foundation for future functional studies and targeted bioprospecting of this Antarctic fungus for novel metabolites with potential biotechnological applications.

## Introduction

*Pseudogymnoascus*, a genus within the *Ascomycota* phylum, is renowned for its capacity to flourish in exceptionally cold conditions, categorizing it as a psychrophilic fungal group (Wang et al. 2015). While these organisms can be found worldwide, they are especially prevalent in polar areas such as the Arctic and Antarctica (Pannkuk et al. 2014). Their remarkable ability to endure and prosper in harsh environments—including low temperatures, intense ultraviolet radiation, and scarce nutrients—has made them a focal point of ecological and biotechnological research (Hassan et al. 2016). Species of *Pseudogymnoascus* found in Antarctica have shown the capability to produce enzymes that function effectively in cold environments, including proteases, lipases, and hydrolases. These enzymes are highly sought after for industrial applications that operate at low temperatures due to their exceptional performance in such conditions (Zucconi et al. 2020). Furthermore, Antarctic *Pseudogymnoascus* strains generate unique bioactive substances, such as sesquiterpenoids and polyketides, which exhibit significant antibacterial and anticancer effects. The varied fungal populations in Antarctic soils and sediments, encompassing *Pseudogymnoascus* species, display robust enzymatic activity and substantial biosynthetic capacity (Shi et al. 2024). This positions them as valuable targets for industrial applications and bioprospecting endeavors to uncover new bioactive molecules (Ogaki et al. 2020). Several species of the genus *Pseudogymnoascus* have been described in this region. Among those isolated from Antarctic soils are *P. antarcticus*, *P. verrucosus*, *P. destructans*, *P. pannorum*, and, more recently, *P. russus* and *P. irelandiae*. The species *P. australis* was subsequently discovered in Antarctic marine sponges. This species is characterized by unique morphological and phylogenetic traits that demonstrate its exceptional adaptation to thrive in cold environments (Villanueva et al. 2021; Childress et al. 2025). Research on *P. australis* has also explored its reactions to UV-B radiation stress. The fungus exhibits effective mechanisms to combat UV-induced damage, as revealed through transcriptomic analysis. This study identified the activation of crucial DNA repair pathways, including base excision repair (BER) and nucleotide excision repair (NER). These mechanisms are vital for maintaining the integrity of the fungal genome when exposed to UV radiation (Teoh et al. 2024). These findings highlight the significant biotechnological potential of these strains. However, to fully harness this potential, it will be essential to implement advanced -omics tools, such as genomics and proteomics, which enable a comprehensive understanding of biological systems by integrating data from different molecular levels (Palazzotto and Weber 2018).

Genome mining has also emerged as an innovative approach to enhance the discovery of new bioactive compounds (Albarano et al. 2020). Research indicates that Antarctic microorganisms can harbor numerous biosynthetic gene clusters (BGCs) for secondary metabolite production, with only approximately 10% of BGCs currently described (Benaud et al. 2021). Culture-dependent techniques rely on the expression of these BGCs to detect, purify, and characterize secondary metabolites. Unfortunately, many biosynthetic pathways remain dormant or cryptic under conventional culture conditions, making them inaccessible for further investigation (Scherlach and Hertweck 2009; Hoshino et al. 2019). Consequently, new methods to activate these silent gene clusters are necessary to improve the exploration and exploitation of microbial strains to discover bioactive metabolites (Ochi and Hosaka 2013). A multi-omics approach that integrates genomics and metabolomics could further enhance the identification and characterization of novel bioactive compounds (Palazzotto and Weber 2018).

Research by Khalil et al. 2014 demonstrates that nitric oxide (NO) influences the expression of BGCs in fungi and bacteria. Their findings reveal that both internally produced and externally introduced NO function as transcriptional regulators, triggering dormant BGCs in *Streptomyces* sp. CMB-M0423. The researchers developed novel NO delivery techniques, including in situ generation through natural chemical cues, lipopolysaccharides (LPS) from Gram-negative bacteria, and external administration via sodium nitroprusside or 1% NO in N_2_ (Khalil et al. 2014). These approaches were considered efficient and economical for accessing valuable microbial compounds.

Dewapriya et al. 2018 introduced a high-yield microbioreactor technique in 24-well plates, named MATRIX, which facilitates rapid and consistent 1 mL cultures of fungal isolates under various conditions (Dewapriya et al. 2018). This method produces numerous reference extracts for strain-specific chemical analysis and can be scaled up to preparative multi-flask/plate formats. In a separate study, Núñez-Montero et al. 2020 examined 34 Antarctic bacterial strains for the production of antibacterial secondary metabolites using LPS elicitation, sodium nitroprusside (SNP), and co-culture techniques. Employing HPLC–QTOF–MS/MS and molecular networks, they observed varying antibacterial activity depending on the strain and medium. Seven strains were chosen for genomic analysis, revealing activated biosynthetic pathways and BGCs associated with actinomycin, carotenoids, and bacillibactin. This approach, supported by genomic and metabolomic analyses, underscores the potential of these tools to unveil chemical diversity in polar microorganisms (Núñez-Montero et al. 2020). However, the systematic application of these elicitation strategies in Antarctic fungi remains underexplored. This study aims to fill that gap by investigating the potential of elicitors such as NO and LPS to activate the production of secondary metabolites in Antarctic fungi, a group with largely unknown biosynthetic potential (Xu et al. 2019). Beyond laborious traditional methods, the integration of genomic and metabolomic analyses offers an efficient pathway to navigate complex chemical spaces and directly target the discovery of novel bioactive Metabolites (Maansson et al. 2016). These metabolites have considerable biotechnological applications, including antifungal, antioxidant, and antitumor agents, as well as natural pigments, biopolymers, and other industrial products (Vaishnav and Demain 2011). Consequently, this research aims to examine the genome and identify the biosynthetic gene clusters of the Antarctic strain *P. australis*. Additionally, it seeks to assess the effects of NO and LPS elicitation on the metabolomic profile using HPLC–QTOF–MS/MS and molecular networking techniques.

## Materials and methods

### Isolation, purification, and characterization of Antarctic fungi

The study utilized samples from (Núñez-Montero et al. 2020). During Antarctic expeditions in 2014 and 2016, fungal specimens were collected from diverse sites across the Antarctic Peninsula, including soil, seawater, and sediment environments. One of the isolates, UA-032-E, was obtained from shoreline sediment at a depth of 0.3 m in the Collins Glacier area, King George Island, Antarctica (67.90665109°S, 69.16353777°E; elevation 0 m). The environmental context of this site is classified as a polar region (broad scale) and shoreline sediment (local scale). The collection date was 1 January 2016. Fungal colonies were isolated and purified through incubation on potato dextrose agar (PDA) at 15°C for up to 30 days. For subsequent experiments, inocula were prepared by cultivating each purified strain on ISP-2 and PDA plates under identical conditions (15°C, 10 days). This methodology ensured the recovery of viable and representative fungal cultures suitable for downstream analyses. The identity and purity of the cultures were confirmed through microscopic observations and genetic assessments, which included amplification and sequencing of the internal transcribed spacer (ITS) molecular marker, following the protocol described by (Pereira et al. 2023). Among the isolated fungi, the strain *P. australis* was selected due to evidence in the scientific literature supporting its remarkable biotechnological potential.

### DNA extraction, library preparation, and whole-genome sequencing

DNA extraction was performed from a pure strain of *Pseudogymnoascus australis* using the DNeasy UltraClean Microbial Kit (QIAGEN, Germany) following the manufacturer’s instructions. DNA was quantified using a Qubit dsDNA HS Assay Kit (Invitrogen, USA), with a minimum concentration of ≥ 50 ng/µL. The integrity of the obtained DNA was visualized on an agarose gel in TAE 1× 0.9% m/v buffer. Samples were sequenced using Illumina and Oxford Nanopore Technologies (ONT). The Illumina library was prepared with 2 × 150 bp paired-end fragments on an Illumina NovaSeq platform. Quality control of the reads was assessed using FastQC v0.11.9 (Anon 2025). Adapters were trimmed and reads filtered using Fastp v0.20.0 (Chen et al. 2018) with the following parameters: --detect_adapter_for_pe -f 12 -F 12. For long-read sequencing, the Rapid Sequencing Kit SQK-RBK004 (ONT) was used for library preparation, and sequencing was performed using an R9.4 flow cell (FLO-MIN106D) on a MinION Mk1C machine with MinKNOW v4.3.7 software. Basecalling was performed using Guppy v5.0.12 in fast mode. Quality control was assessed using Nanoplot v1.40.0. Adapters were trimmed with Porechop v0.2.4, and sequences with a quality ≥ 10 were retained for further analysis using Nanofilt v2.8.0 (De Coster et al. 2018).

### Hybrid genome assembly

Following the assembler’s command list, a hybrid assembly between Illumina paired-end and Nanopore reads was performed using MaSuRCA (https://github.com/alekseyzimin/masurca) (Zimin and Salzberg 2020). Subsequently, POLCA was used to improve consensus accuracy in the assembly using Illumina reads with the command polca.sh -a assembly.fasta-r ‘pe_R1.fa pe_R2.fa’ -t 32 -m 32G. After polishing the assembly, the SAMBA scaffolder was used to improve assembly contiguity and fill gaps using the Nanopore long reads with the command: samba.sh -r polished_assembly.fasta -q nanopore_reads.fastq.gz -t 32 (Zimin and Salzberg 2022). The assembly quality was evaluated using QUAST (https://github.com/ablab/quast), considering metrics such as N50 and the total length of the assembled genome (Gurevich et al. 2013).

### Repeat masking, gene prediction, and functional and secondary metabolism annotation

Repeat masking and gene prediction were conducted using advanced tools to ensure accurate annotation, following the pipeline outlined by (Aylward et al. 2024) as a reference. RepeatModeler v2.0.1 (Flynn et al. 2020) was utilized to generate a custom library of repeat families specific to each assembly. These libraries were then applied for soft masking of the assembly (v2.1) with RepeatMasker open-4.1.7.-p1. Gene prediction was subsequently carried out on the masked assembly (v3.0) (Stanke et al. 2006) using Funannotate predict, which integrates multiple ab initio prediction tools, including Augustus v3.4.0, GeneMark-ES (Ter-Hovhannisyan et al. 2008), Glimmer v3.0.4 (Delcher et al. 2007), and SNAP (Korf 2004). In addition to these predictors, protein evidence was incorporated through BLAST alignments against the UniProt database (Bateman et al. 2023), while tRNA genes were identified using tRNAscan-SE v2.0.12 (Lowe and Eddy 1997). This comprehensive approach ensured a robust annotation of the assembly. The functional annotation of predicted genes was performed by comparing them against various annotation databases. This process involved using stand-alone tools such as InterProScan v5.72–103 (Jones et al. 2014; Blum et al. 2021), EggNOG-mapper v2.1.112 (Cantalapiedra et al. 2021), antiSMASH fungal v7.1.12 performed with relaxed detection (Blin et al. 2021), and Phobius v1.01 (Käll et al. 2007). The resulting annotations were consolidated into a single file using the Funannotate annotate pipeline, which also included comparisons of predicted proteins with the dbCAN v13.0 database of carbohydrate-active enzymes (CAZymes) (Yin et al. 2012) and the MEROPS v12.0 protease database (Rawlings et al. 2014). Additionally, the mating-type loci were identified in this genome through local BLASTn and tBLASTn searches using MAT gene or protein sequences from closely related species. To analyze the secondary metabolism, antiSMASH analysis was performed using antiSMASH version 8.0.1 with relaxed detection strictness, employing default fungal parameters for BGC prediction (Blin et al. 2025). BiG-SCAPE analysis was performed on the antiSMASH-predicted BGCs using BiG-SCAPE (v2.0.0-beta.7) with Pfam-A.hmm profiles. The run was conducted at the region level with glocal alignment, including singletons, using a similarity cutoff of 0.5 and mixed weights (Navarro-Muñoz et al. 2019). The graphical analyses and visualizations presented in this study were performed using the Python programming language (version 3.8). For the generation, customization, and export of figures, the following packages were employed: pandas (v1.3.3) for data manipulation and structuring, matplotlib (v3.4.3) as the base library for creating static plots, seaborn (v0.11.2) to enhance the aesthetic style of visualizations, and plotly (v5.3.1) for generating interactive graphs, while the circular genome visualization was performed using Circus v0.69-8 (Naquin et al. 2014).

### Screening of culture conditions for elicitation of secondary metabolites

A mycelial fragment of the fungal strain, obtained from cultures grown on PDA medium, was inoculated into 1.5 mL of each of twelve different media using a micro-bioreactor system (Applikon Biotechnology, Delft, The Netherlands). An uninoculated well for each medium was kept as a negative control. Cultures were incubated at 15°C and 190 rpm for 7 days. The extraction and detection of secondary metabolites were conducted as explained in the next section. Five culture media were selected (M2, IMA, YES, ISP-4, and CG4) containing different sources of C and N as shown in Table 1 as more suitable for secondary metabolite production. The cultures were repeated using selected media as previously described under two elicitation conditions: sodium nitroprusside (SNP 2 μM) as a nitric oxide donor and LPS from Escherichia coli O55:B5 (Merck LPS 0.6 nM). In addition, sterile water or spent SNP (exposed to light for 10 days) was used as an untreated control for LPS and SNP, respectively. After 7 days of incubation at 15°C and 190 rpm, extraction and detection of secondary metabolites were performed for each treatment.

**Table 1.** Culture media composition.

Medium	Components (per litre)
M2	Mannitol (40 g), Maltose (40 g), Yeast extract (10 g), K_2_HPO_4_ (2 g), MgSO_4_·7H_2_O (0.5 g), FeSO_4_·7H_2_O (0.01 g)
IMA	Yeast extract (4 g), Malt extract (10 g), Glucose (4 g), Mannitol (40 g)
YES	Sucrose (150 g), Yeast extract (20 g), MgSO_4_·7H_2_O (0.5 g), ZnSO_4_·7H_2_O (0.01 g), CuSO_4_·5H_2_O (0.005 g)
ISP-4	Starch (10 g), CaCO_3_ (2 g), (NH_4_)_2_SO_4_ (2 g), K_2_HPO_4_ (1 g), MgSO_4_·7H_2_O (1 g), NaCl (1 g), FeSO_4_·7H_2_O (1 mg), MnCl_2_·7H_2_O (1 mg), ZnSO_4_·7H_2_O (1 mg)
CGA	Glycerol (30 g), Peptone (2 g), K_2_HPO_4_ (1 g), NaCl (1 g), MgSO_4_·7H_2_O (0.5 g), Trace solution (5 mL): CaCl_2_·2H_2_O (3 g/L), MnSO_4_ (0.2 g/L), ZnCl_2_ (0.1 g/L), CuSO_4_·5H_2_O (0.025 g/L), Na_2_B_4_O_7_·10H_2_O (0.02 g/L), CoCl_2_ (0.004 g/L), (NH_4_)_6_Mo_7_O_24_·4H_2_O (0.01 g/L)

### Extraction and detection of secondary metabolites by LC–MS/MS

Extraction of each culture was performed in situ by adding 2 mL of ethyl acetate (EtOAc). After [min of incubation at 190 rpm, the organic phase containing secondary metabolites was collected, dried under N_2_ airflow, and reconstituted in 20 μL of methanol for LC–QTOF (+)–MS/MS analysis. Aliquots (1 μL) of the dried resuspended fraction were analyzed on an Agilent 6545 Q-TOF LC/MS equipped with an Agilent 1290 Infinity II UPLC system. Chromatographic separation was carried out on a Zorbax C8 RRHD column (1.8 μm, 2.1 × 50 mm; Agilent). Elution used a 2.5-min linear gradient at 0.417 mL/min, from 90% water/10% acetonitrile to 100% acetonitrile, enabling efficient elution of increasingly hydrophobic analytes. Mass spectrometric detection was performed under conditions optimized to favor protonation. Under these acquisition settings, [M+H]+ adducts consistently represented the most abundant and reproducible features across all samples. Downstream analyses therefore focused on [M+H]+ ions to ensure consistency and comparability among strains. A collision energy of 20 eV was applied to induce fragmentation of precursor ions and generate product ion spectra suitable for structural elucidation.

### LC–QTOF–MS/MS metadata analysis and annotation of secondary metabolites

The MZmine v4.0.3 program (Kim et al. 2021) was used to preprocess the relevant adduct ions [M+H]+ for Feature-Based Molecular Networking (FBMN). The parameters were kept for all strains processed and are as follows: Ion Mode = Positive Absolute Intensity; Noise Threshold = MS1: 1.0E5, MS2: 1.0E2; Minimum Feature Height = 3.0E2; Crop Retention Time = autorange; Feature list blank subtraction of the spectra of the media and blanks used. The molecular networks were created using the GNPS FBMN analysis and annotation tool (Wang et al. 2016) for all strains. The following parameters were kept consistent: Precursor Ion Mass Tolerance = 0.02; Fragment Ion Mass Tolerance = 0.02. All datasets used in this project are available in the GNPS repository: ftp://massive.ucsd.edu/MSV000085261/. The SIRIUS (Dührkop et al. 2019) program tools were utilized to perform *in silico* metabolite prediction and annotation. SIRIUS, ZODIAC, CSI:FingerID, and CANOPUS tools were used for this process. For CSI:FingerID, all available databases were selected for compound identification, and CANOPUS was used for natural product class predictions according to NPClassifier (Kim et al. 2021). Visualization and data processing were done on Cytoscape v3.7.1 (The Cytoscape Consortium, California, USA) and included the data from both GNPS FBMN annotation and SIRIUS *in silico* predictions to facilitate the analysis of all features.

## Results

### Sequencing and annotation of the *Pseudogymnoascus australis* UA-032-E

**Figure 1.**
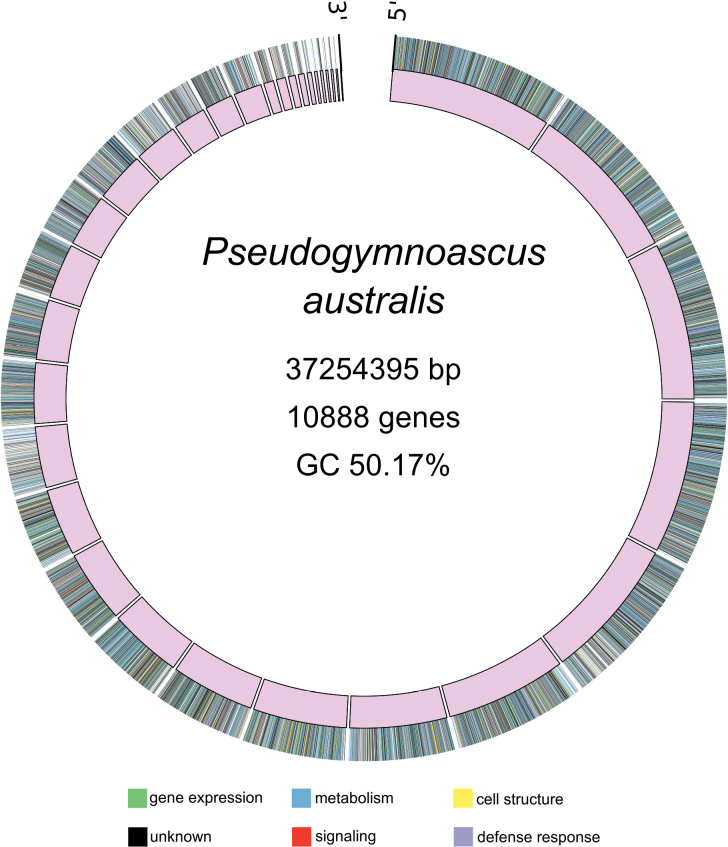
Genome assembly visualization of *P. australis* UA-032-E using a Circus plot. The image depicts the genomic structure and organization, comprising 33 contigs. Colors on the outer ring denote genes with unidentified functional elements within the genome, according to the legend. Functional classification of genes was conducted using the Clusters of Orthologous Groups (COGs) database categories, thus offering a comprehensive view of the genome architecture and organization of *P. australis* UA-032-E. Circular representation was done using Circos v0.69-8.

The complete genome of the fungal strain *P. australis* UA-032-E was sequenced using Nanopore and Illumina technologies, resulting in a hybrid assembly. The resulting sequences were evaluated according to quality parameters, and it was determined that the genome has over 96% completeness. Additionally, the genome assembly consists of 33 contigs, with the largest contig measuring 3,190,541 bp and a total genome length of 37,254,395 bp (Fig. 1). The genome assembly of *Pseudogymnoascus australis* UA-032-E demonstrates high continuity, as reflected by an N50 of 1.91 Mbp and an N90 of 0.97 Mbp, with L50 and L90 values of 7 and 18, respectively. The overall GC content was 50.16%. The assembled genome has been deposited in the NCBI database under the accession number JBLVTM000000000. Phylogenetic analysis based on concatenated ITS, RPB2, and Tef1 sequences strongly supports the classification of UA-032-E within the *P. australis* clade, with high statistical support (99% ML bootstrap / – BI posterior probability), clearly distinguishing it from related species such as *P. griseus*, *P. papyriferae*, and *P. shaanxiensis* (Suppl. material 1: section S1). Functional annotation identified 10,800 protein-coding genes, along with 48 rRNA and 199 tRNA genes. Additionally, 2,923 proteins were predicted to contain transmembrane domains, and 9,981 proteins were associated with PFAM domains, highlighting the functional diversity and genomic completeness of this Antarctic fungal strain.

### Analysis of biosynthetic gene clusters of the *P. australis* UA-032-E

**Figure 2.**
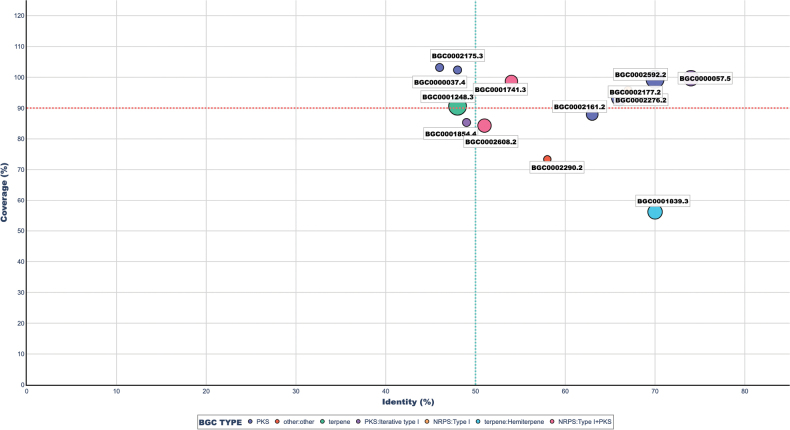
Comparative analysis of BGCs identified in *P. australis* UA-032-E using antiSMASH and annotated by similarity to the MIBiG database. The X-axis shows sequence identity (%) and the Y-axis shows coverage (%) based on BLAST alignments between the predicted BGCs and their most similar known counterparts in MIBiG. Each point represents an individual BGC, where the color indicates the biosynthetic type (e.g., PKS, NRPS, terpene, or hybrids), the point size corresponds to the BLAST score (larger size = stronger similarity), and the symbol shape reflects the confidence level assigned to the annotation. Dashed lines represent selection thresholds: 50% identity (vertical turquoise line) and 90% coverage (horizontal red line), which are considered the minimum criteria to classify a BGC as highly similar to a previously characterized cluster. BGCs located in the upper right quadrant exhibit both high identity and high coverage, suggesting strong functional similarity to known clusters.

The genome analysis of *P. australis* UA-032-E using the antiSMASH tool led to the prediction of a total of 44 BGCs. Among them, 13 BGCs showed significant similarity to previously characterized entries in the MIBiG database, based on their BLAST score, percentage identity, coverage, and E-value. As shown in Fig. 2, which graphically represents the identity and coverage values of each annotated BGC, those located in the upper right quadrant meet both threshold criteria (≥50% identity and ≥90% coverage), indicating a high degree of functional similarity with reference BGCs. Notable examples (Table 2) include clusters associated with the production of geodin (BGC0002592.2; 70% identity, 99.39% coverage), F9775A/F9775B/orsellinic acid (BGC0000057.5; 74%, 99.70%), and ustilaginoidin N/O/M/A (BGC0002177.2; 66%, 93.23%). Conversely, BGCs such as BGC0000037.4 (cichorine) and BGC0002175.3 (YWA1) exhibited coverage values exceeding 100% (103.18% and 102.37%, respectively) but only moderate identity (46% and 48%), suggesting the presence of structurally conserved variants with sequence divergence. In contrast, BGC0001839.3 (squalestatin S1) showed high identity (70%) but low coverage (56.16%), placing it outside the upper threshold.

**Table 2.** BGCs identified in *P. australis* UA-032-E using antiSMASH and their similarity to MIBiG entries.

MIBiG Clusters	Source Compounds	Type	Total Score	Best Identity	Best Coverage	E-value
BGC0000037.4	cichorine	PKS	2122.00	46%	103.18%	0.0
BGC0002290.2	biscognienyne B / eutypinic acid	other:other	320.00	58%	73.37%	2.28e-43
BGC0001248.3	clavaric acid	terpene	700.00	48%	90.60%	2.61e-245
BGC0000057.5	F9775A/F9775B/orsellinic acid	PKS:Iterative type I	4033.00	74%	99.70%	2.42e-184
BGC0002276.2	choline	NRPS:Type I	1701.00	67%	95.81%	0.0
BGC0001854.4	tricholignan A	PKS:Iterative type I	3623.00	49%	85.32%	0.0
BGC0001839.3	squalestatin S1	terpene:Hemiterpene	957.00	70%	56.16%	1.62e-162
BGC0001741.3	phyllostictine A/B	NRPS:Type I+PKS	3639.00	54%	98.66%	2.74e-120
BGC0002177.2	ustilaginoidin N/O/M/A	PKS	4095.00	66%	93.23%	6.55e-178
BGC0002161.2	scytalone/T3HN	PKS	2821.00	63%	87.94%	1.95e-107
BGC0002592.2	geodin	PKS	5323.0, 2596.0	70%	99.39%	8.42e-251
BGC0002175.3	YWA1	PKS	1979.00	48%	102.37%	0.0
BGC0002608.2	ochratoxin A	NRPS:Type I+PKS	412.00	51%	84.31%	1.06e-139

**Figure 3.**
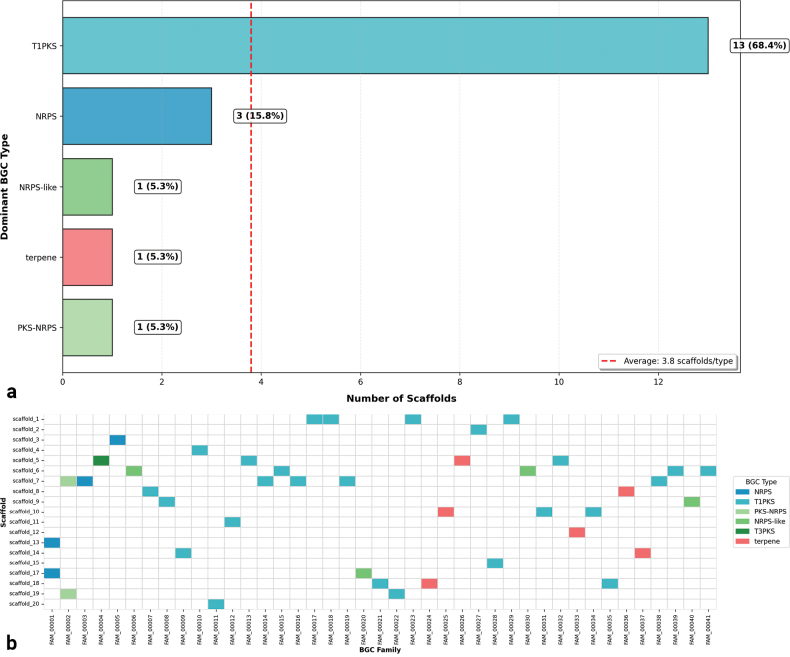
Comprehensive analysis of BGC distribution in the genome of *P. australis* UA-032-E. a Distribution of dominant BGC types across scaffolds, revealing the predominance of T1PKS systems. Color coding represents biosynthetic types: NRPS (blue), T1PKS (light blue), PKS-NRPS hybrids (light green), NRPS-like (green), T3PKS (dark green), and terpene (red). Bar values indicate the number of scaffolds and corresponding percentage. The dashed red line marks the average number of scaffolds per type. This distribution suggests specialized genomic regions favoring particular biosynthetic pathways with significant representation of both modular systems and specialized metabolism, highlighting the functional organization of the organism’s biosynthetic potential. b Heatmap displaying BGCs grouped by family (vertical axis) and their genomic location by scaffold (horizontal axis). BiG-SCAPE analysis identified 44 BGCs classified into 41 families, with only two families containing multiple members, indicating high structural diversity.

Following the identification of 44 BGCs in the genome of *P. australis* UA-032-E using antiSMASH, comparative analysis was performed using BiG-SCAPE to group them into functional families based on structural and sequence similarities. This large-scale comparative approach allows for the inference of evolutionary relationships and the identification of potentially novel biosynthetic pathways, facilitating the discovery of bioactive secondary metabolites. The predicted BGCs encompassed a broad range of biosynthetic types: T1PKS, 20 clusters, followed by NRPS, 8 clusters, terpenes, 5 clusters, and several hybrid or variant types, including NRPS-T1PKS, 4 clusters (Fig. 3a). The analysis revealed a total of 41 gene cluster families, of which 39 were singletons, indicating that most BGCs shared low similarity with each other and likely represent unique biosynthetic pathways (Fig. 3b). Two multi-member BGC families, FAM_00001 and FAM_00002, suggest minimal genetic redundancy in *P. australis*, indicating a limited biosynthetic repertoire (Suppl. material 1: section S2). Notably, the BGC family FAM_00002 displays a unique genomic arrangement, with identical assemblies found on three separate contigs. This family comprises three NRPS-type BGCs with a modular structure featuring the canonical A–T–C domain triad, which includes PF00501 (AMP-binding; adenylation domain), PF00550 (phosphopantetheine attachment site; thiolation domain), and PF00668 (condensation domain). This configuration is characteristic of NRPS systems that facilitate substrate activation and peptide bond formation. These clusters are predicted to produce small cyclic peptides with 2 to 4 residues, incorporating various combinations of aromatic (e.g., phenylalanine, tyrosine, tryptophan) and aliphatic (e.g., valine, isoleucine) amino acids. Interestingly, two of these NRPS closely resemble BGC0000357.5 from *Penicillium rubens*, a known cluster encoding diketopiperazine synthases that generate cyclopeptides with the general structure cyclo-(D-aa–L-aa–D-aa–L-aa). Conversely, the BGC family FAM_00001, with identical assemblies on two distinct contigs (scaffold_17.region002 and scaffold_13.region001), encodes a highly conserved iterative T1PKS involved in synthesizing 1,3,6,8-tetrahydroxynaphthalene (THN), a crucial precursor in the DHN-melanin biosynthetic pathway. Comparative analysis using the MIBiG database reveals that FAM_00001 shares 81% sequence identity with BGC0001258.3 from *Glarea lozoyensis*, a known THN-producing cluster. Functional annotation of its biosynthetic genes shows a minimal yet catalytically complete PKS module, including PF00109 (β-ketoacyl synthase N-terminal domain), PF00083 (β-ketoacyl synthase C-terminal domain), PF00698 (acyltransferase), PF16073 (ACP transacylase), PF00550 (phosphopantetheine attachment site; ACP), PF14765 (dehydratase domain), and PF00975 (thioesterase domain). This setup supports iterative polyketide chain elongation through decarboxylative condensations of malonyl-CoA units (facilitated by the AT domain with a conserved GQGxQ motif), intermediate shuttling by the ACP, and product release by the TE domain, ultimately leading to the formation of the naphthalene scaffold through spontaneous cyclization.

### Metabolomic profiling and structural elucidation of compounds from *P. australis* UA-032-E under varied growth conditions

**Figure 4.**
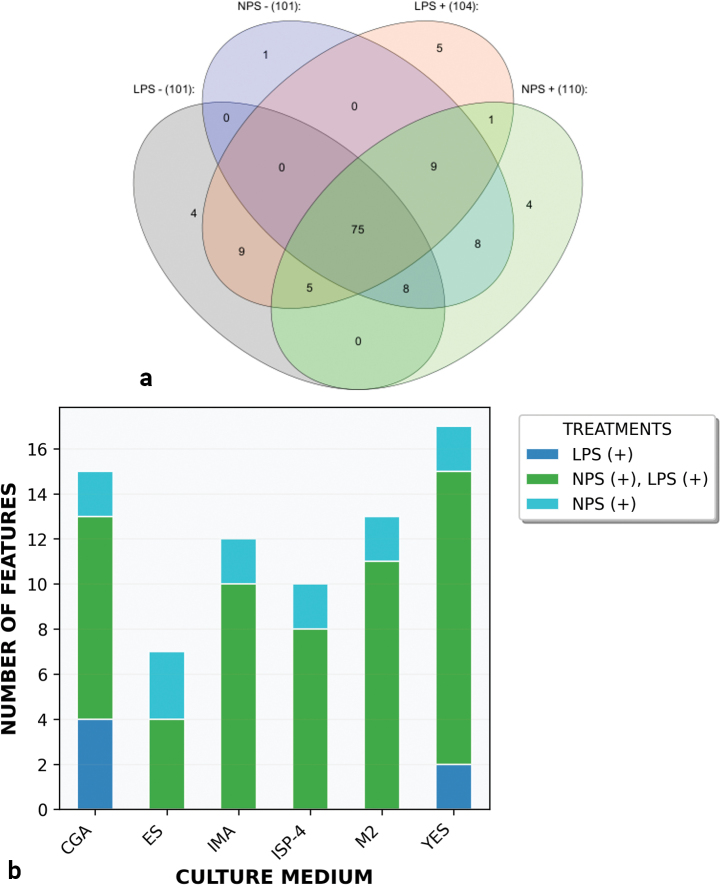
Venn diagram showing the distribution of compounds identified in the treatments LPS(−), LPS(+), NPS(−), and NPS(+). a A common core of 75 features is present across all four treatments, while other areas of the diagram reveal compounds specific to each treatment or combination of them. Positive treatments (LPS+ and NPS+) share a greater number of compounds with each other compared to the negative treatments, suggesting potential differences in the metabolic responses induced by the experimental stimuli. b The graph represents the number of features generated with respect to the growth medium and the treatment applied to the samples. A stacked bar chart was used to visualize these variations across six distinct media: CGA, ES, IMA, ISP-4, M2, and YES. The treatments evaluated included combinations of NPS(−), NPS(+), LPS(−), LPS(+), and NPS(+)/LPS(+).

Metabolomic profiling of *P. australis* was performed using LC–QTOF–MS/MS, with subsequent data analysis leveraging the GNPS Feature-Based Molecular Networking platform and the SIRIUS/CANOPUS computational suite. This integrated strategy enabled extensive annotation of biologically relevant molecular features by combining spectral matching with *in silico* fragmentation prediction. A Venn diagram (Fig. 4a) illustrates the overlap of metabolic features across the four experimental treatments [LPS(−), LPS(+), NPS(−), NPS(+)], revealing a core set of 75 MS features consistently present under all conditions. Results are interpreted at the level of putatively annotated MS features rather than fully validated metabolites. Further analysis of feature distribution across culture media (CGA, IMA, ISP-4, M2, YES) and elicitation conditions [NPS(−), NPS(+), LPS(−), LPS(+), and combined NPS(+)/LPS(+)] is presented in a bar chart (Fig. 4b). The combined elicitation treatment (NPS+/LPS+) yielded the highest number of detected features. Among culture media, YES and CGA supported the greatest diversity of secondary metabolite-related features, indicating their particular suitability for eliciting a broad spectrum of metabolic responses in *P. australis*.

### Metabolic profile and diversity of secondary metabolites in *P. australis* UA-032-E

**Figure 5.**
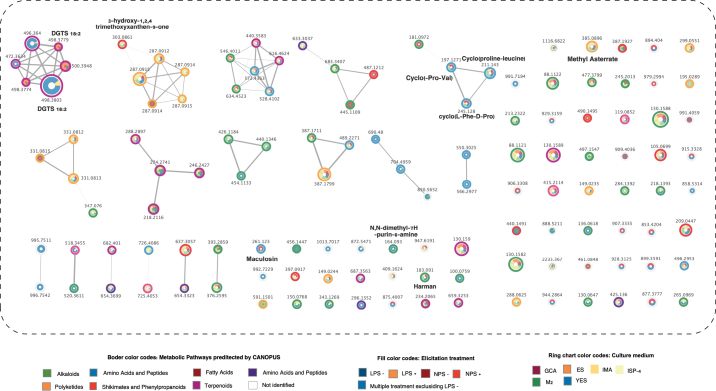
Molecular networking of adduct ions produced by *P. australis* UA-032-E cultivated under different conditions, analyzed using HPLC–QTOF–MS/MS, with metadata processed through the GNPS platform and SIRIUS/CANOPUS. Border color refers to the metabolic pathway predicted by CANOPUS: green for alkaloids, yellow for polyketides, blue for amino acids and peptides, red for shikimates and phenylpropanoids, burgundy for fatty acids, pink for terpenoids, purple for carbohydrates, and white for not identified. Fill colors represent the elicitation treatment at culture: blue for LPS−, grey for LPS+, yellow for NPS−, orange for NPS+, and green for multiple treatments excluding LPS−. Ring chart colors refer to the culture medium used: red for CGA, orange for ES, light orange for IMA, yellow for ISP-4, green for M2, and blue for YES. The diameter of the nodes/features is proportional in size to the intensity of the precursor in the initial sample. The width of the edges represents the cosine score with values between 0.5 and 5.0.

The chemical composition of *P. australis* was characterized through a comprehensive metabolomic analysis employing LC–QTOF–MS/MS. Compound annotation was achieved by integrating two complementary computational approaches. First, molecular networking via the GNPS platform facilitated the dereplication of known metabolites by comparing acquired MS/MS spectra against public spectral libraries and grouping related molecules based on spectral similarity (Fig. 5). Second, to annotate novel or library-unmatched features, the SIRIUS computational suite was used, which combines CSI:FingerID for molecular structure prediction via fragmentation tree analysis and CANOPUS for predicting compound class labels directly from MS/MS spectra without requiring a reference library. This combined strategy allowed for the comprehensive annotation of both known and previously unreported metabolites in the extract.

The GNPS molecular network revealed a significant presence of different metabolites (Table 3). Notably, diacylglyceryl-N,N,N-trimethylhomoserine (DGTS 18:2) was identified, producing a characteristic fragment ion at *m/z* 498.3800. This fragment corresponds to the monoacyl form, comprising a glycerol backbone, homoserine, and a single linoleic acid (C18:2) chain, which is consistent with the established fragmentation pattern for DGTS-type betaine lipids. Furthermore, the alkaloid harman was annotated and detected as its protonated adduct [M+H]^+^ at *m/z* 183.0910. The high spectral similarity to previously reported data supports its confident identification and indicates the presence of β-carboline alkaloids in the metabolic profile of this fungus. Another identified compound was N^6^,N^6^-dimethyladenine (N,N-dimethyl-7H-purin-6-amine), observed as a protonated molecule [M+H]^+^ at *m/z* 164.0930, matching its exact theoretical mass. This methylated purine derivative is a known precursor and has been implicated in fungal cytokinin biosynthesis pathways. Recent studies have established that certain fungal species, including *Claviceps purpurea*, biosynthesize bioactive cytokinins, such as trans-zeatin, via dedicated de novo pathways (Hinsch et al. 2015). Furthermore, three diketopiperazine (DKP) cyclic dipeptides were annotated based on precursor masses matching their theoretical values in [M+H]^+^ mode. The compounds cyclo(L-Phe-L-Pro) (*m/z* 245.1280), cyclo(L-Val-L-Pro) (*m/z* 197.1281), and cyclo(L-Pro-L-Leu) (*m/z* 211.1437) were identified. These metabolites, characterized by their stable diketopiperazine ring structure, are commonly produced by fungi. Their annotation is corroborated by the GNPS molecular network, where each compound formed a distinct node. The identification of cyclo(L-Val-L-Pro) is consistent with prior reports from fungi such as *Penicillium chrysogenum* and *Schizophyllum commune*. Similarly, cyclo(L-Pro-L-Leu) has been previously isolated from various fungal species, including the endophytic fungus *Aspergillus terreus* and marine-derived *Pseudofusicoccum* sp. (Bach et al. 2022). Furthermore, the cyclic dipeptide cyclo(L-Pro-L-Leu) was identified with an observed [M+H]^+^ ion at *m/z* 211.1430. This diketopiperazine has been previously reported in various fungi, including the endophytic strain *Aspergillus terreus* GX-3 and the marine-derived fungus *Pseudofusicoccum* sp., often co-occurring with other bioactive DKPs (Ma et al. 2021; Alves et al. 2025). The presence of cyclo(L-Pro-L-Leu), along with cyclo(L-Phe-L-Pro) and cyclo(L-Val-L-Pro), is robustly supported by the GNPS molecular network, wherein each metabolite forms a distinct node connected by spectral similarity to known library matches. This consolidated evidence confirms the production of these characteristic fungal diketopiperazines by *P. australis*.

**Table 3.** Metabolites annotated via GNPS from *P. australis* under different treatments.

Feature ID	Medium	Treatment	GNPS Annotation	Precursor Mass
1	YES, ES	NPS (+)	DGTS 18:2	498.3774
2	YES, ES	NPS (+)	DGTS 18:2	498.3779
3	YES, IMA	LPS (−), NPS (−), NPS (+), LPS (+)	DGTS 18:2	498.3803
4	YES, M2, CGA, IMA	LPS (−), NPS (−), NPS (+), LPS (+)	Harman	183.0910
5	YES	LPS (−), NPS (−), NPS (+), LPS (+)	N,N-dimethyl-7H-purin-6-amine	164.0930
6	YES, M2, CGA, IMA	LPS (−), NPS (−), NPS (+), LPS (+)	cyclo(L-Phe-D-Pro)	245.1280
7	YES, M2, CGA, IMA	LPS (−), NPS (−), NPS (+), LPS (+)	cyclo(L-Val-L-Pro)	197.1271

Annotations generated by the SIRIUS platform further highlighted a diverse array of peptide and polyketide derivatives (Table 4). Notably, the cyclic diketopiperazine maculosin [cyclo(L-Pro-L-Tyr)] was identified, with an observed [M+H]^+^ ion at *m/z* 261.1230, matching its exact theoretical mass. This compound, previously isolated from fungi such as *Alternaria alternata*, is recognized for its antimicrobial properties. Additionally, the polyketide-derived metabolite methyl asterrate was annotated among the detected secondary metabolites (Castaldi et al. 2022). The methyl ester derivative methyl asterrate was tentatively annotated based on an observed [M+H]^+^ ion at *m/z* 385.0896. This fungal secondary metabolite, previously reported in *Geomyces* sp., has been associated with inhibitory activity against *Aspergillus fumigatus* (Liu et al. 2016). Similarly, the oxygenated xanthone derivative 3-hydroxy-1,2,4-trimethoxyxanthen-9-one was identified with a precursor mass of *m/z* 303.0861 [M+H]^+^, matching its theoretical mass. This class of compounds is commonly produced by fungi, including genera such as *Penicillium*, *Xylaria*, and *Paraphaeosphaeria* (Ge et al. 2006; Shao et al. 2008; Wang et al. 2018).

**Table 4.** Metabolites annotated via SIRIUS from *P. australis* under different treatments.

Feature ID	Medium	Treatment	Sirius Name	Precursor Mass
1	YES,M2,CGA,IMA	LPS (-),NPS (-),NPS (+),LPS (+)	Maculosin	261.123
2	YES,M2,CGA,IMA,ISP-4	LPS (-),NPS (-),NPS (+),LPS (+)	Methyl Asterrate	385.0896
3	YES,M2,IMA,ISP-4	LPS (-),NPS (-),NPS (+),LPS (+)	3-hydroxy-1,2,4-trimethoxyxanthen-9-one	303.0861

## Discussion

The fungal genus *Pseudogymnoascus* is renowned for its remarkable ability to thrive in extreme conditions, particularly in polar regions characterized by low temperatures, intense ultraviolet radiation, and limited nutrients. These demanding environments have fostered the evolution of unique metabolic and physiological adaptations in these organisms. Recent studies have demonstrated that *Pseudogymnoascus* produces secondary metabolites with considerable biotechnological applications, including pharmaceutical and textile use (Satriawan et al. 2024). A notable example is the research conducted by Antipova et al. (Antipova et al. 2023), which explored the genus *Pseudogymnoascus* and uncovered its exceptional biosynthetic capacity for generating bioactive compounds. Their investigation identified the production of 16-membered trilactone macrolides, (+)-macrosphelides A and B, in the Arctic strains VKM F-4518 and VKM F-4519, with macrosphelide A emerging as a promising candidate for cancer therapy. Moreover, a comprehensive analysis of metabolites produced by the fungus *Pseudogymnoascus* sp. HSX2#-11, guided by molecular networking techniques, resulted in the isolation of a new polyketide, pseudophenone A, along with six previously identified analogues. The structure of this novel compound was elucidated through extensive spectroscopic analysis and single-crystal X-ray diffraction. The compound, named pseudophenone A, is composed of a dimer of diphenyl ketone and diphenyl ether, and to our knowledge, only one analog of this structural class has been previously reported. Both pseudophenone A and compound 2 displayed antibacterial activity against various bacterial strains (Shi et al. 2021). These discoveries emphasize the effectiveness of molecular networking as a powerful tool for identifying new bioactive compounds in psychrophilic fungi, thus supporting the methodology employed in our study. Furthermore, they underscore the substantial metabolic potential of *Pseudogymnoascus* spp. as a valuable resource for future investigations aimed at exploring secondary metabolites with biotechnological applications (Yoshinaga et al. 2024).

*P. australis*, a species discovered in Antarctic marine sponges, exhibits distinct morphological and phylogenetic traits that set it apart from other members of its genus (Villanueva et al. 2021). Teoh et al. (Teoh et al. 2024) conducted a study examining the transcriptomic responses of an Arctic strain to UV-B radiation. The strains were grown on nylon membranes atop Czapek-Dox agar at 15°C in dark conditions for 10 days until reaching exponential phase. They were then subjected to a UV-B dose of 6.1 kJ m^−2^ d^−1^ for 130 minutes and examined under light after 0, 2, and 6 hours. RNA-Seq analysis (40 million reads per sample) revealed an increase in radiation repair (RAD) genes and base excision repair (BER) and nucleotide excision repair (NER) genes. In contrast, the photoreactivation gene (PHR1) showed decreased expression. Additionally, a reduction in oxidoreductase activity was observed as a stress adaptation mechanism. This pioneering analysis sheds light on *P. australis* DNA repair mechanisms, offering insights into its resilience to extreme environments and UV-induced damage. The complete genome sequencing of *P. australis* marks a significant advancement in our comprehension of its genetic and enzymatic potential. This achievement enables the precise identification of crucial genes and exploration of related biosynthetic pathways, underscoring the organism’s substantial biotechnological value.

To date, only one study has reported the identification of secondary metabolites in *P. australis* from the Arctic region using the antiSMASH tool. Antipova et al. (Antipova et al. 2023) reported 32 BGCs in the genome of strain VKM F-4518, including 11 T1PKS, 9 NRPS, 1 T3PKS, 1 mixed (T1PKS, NRPS), 7 fungal RiPP, and 3 terpene cyclases (T). In strain VKM F-4519, 17 clusters were identified: 8 T1PKS, 6 NRPS, 1 hybrid (T3PKS and NRPS), and 2 fungal RiPP, with similarities to known pathways ranging from 12% to 100%. The genomic analysis of *P. australis* UA-032-E identified 44 BGCs, of which 13 showed high similarity to known clusters in MIBiG (e.g., geodin, ustilaginoidins). Among them, the most notable are 6 PKS, 2 iterative type I PKS, 1 type I NRPS, 2 NRPS–PKS hybrid clusters, 1 classified as other, 1 terpene, and 1 hemiterpene cluster. When comparing *P. australis* from Arctic regions with the Antarctic-origin *P. australis* from our study, both exhibit a clear tendency to produce PKS- and NRPS-type BGCs. The high abundance of PKS and NRPSBGCs in fungi reflects their evolutionary adaptation to diverse ecological niches, enabling the production of complex secondary metabolites for defense, competition, and symbiosis (Schümann and Hertweck 2007; Minami et al. 2020; Allen et al. 2024). This metabolic diversity is further shaped by gene duplication, environmental pressures, and host interactions (Bergmann et al. 2007; Collemare et al. 2008).

BiG-SCAPE analysis, used to group BGCs based on structural and functional similarity, identified two notable GCFs: FAM_00002, predicted to encode an NRPS for cyclic peptide biosynthesis, and FAM_00001, an iterative PKS putatively involved in THN biosynthesis. The remaining 39 BGCs did not cluster into families (i.e., were singletons), a common outcome reflecting high biosynthetic diversity. This pattern of few conserved families alongside abundant singletons suggests that, like other fungal species such as *Xylaria hypoxylon* and *Aspergillus* spp., *P. australis* possesses a reservoir of unique biosynthetic potential. This significantly expands the known chemical space available for the discovery of novel bioactive secondary metabolites (Yang et al. 2020). Therefore, beyond characterizing genomic diversity, BiG-SCAPE is instrumental in prioritizing novel clusters, particularly those within GCFs, for functional studies or drug discovery efforts.

Metabolomic analysis and elicitation treatments using various media and substances like LPS and SNP resulted in the induction of 75 features, which were identified through LC–QTOF–MS/MS. This outcome demonstrates the efficacy of this approach in generating new compounds. A study by Khalil et al. (Khalil et al. 2014) demonstrated that LPS serves as a natural chemical stimulant capable of altering secondary metabolic expression in fungal species such as *Penicillium* sp. and *Aspergillus* sp. The research indicated that LPS treatment activated secondary metabolites in 15% of the studied strains, with an optimal concentration of 0.6 ng/mL. Additionally, this activation enhanced the production of bioactive substances, including pseurotins and neoasterriquinones. These findings indicate that LPS stimulation can unlock the molecular potential of fungal strains previously inactive under standard cultivation conditions. Research on NO has shown its considerable potential and identified several crucial mechanisms underlying its effects. NO functions as a signaling molecule regulating vital processes in fungi, with nitrate reductase producing it in response to environmental stressors or during fungal developmental phases. Moreover, NO plays a critical role in neutralizing nitrosative stress and promoting biosynthetic pathways for secondary metabolites, thereby enhancing the expression of genes involved in producing cryptic metabolites (Cánovas et al. 2016; Zhao et al. 2020). The set of 75 synthesized features displays a broad range of structural variations. The use of LPS and SNP elicitors together, irrespective of the culture medium, resulted in a greater production of compounds than when each was used separately (Fig. 3a). These results are consistent with those of Núñez-Montero et al. (Núñez-Montero et al. 2020), who found through MS/MS metabolomics and genome mining that combining elicitors can trigger dormant metabolic pathways and enhance metabolite diversity in Antarctic bacteria. The synthesis of these molecules can be influenced by various environmental conditions and the presence of elicitors that trigger specific metabolic pathways. Biotic elicitors, such as fungi and bacteria, have been identified as key agents in the induction of secondary metabolite biosynthesis. Bhaskar et al. (Bhaskar et al. 2021) reported that exposure to *Aspergillus niger* increased thiophene production by 85%, while *Botrytis* spp. promoted a 26-fold increase in sanguinarine synthesis. Additionally, extracts from *Haematococcus pluvialis* enhanced betalain production by 2.28-fold. Regarding chemical elicitors, methyl jasmonate (MeJA) has been identified as a potent regulator of secondary metabolite synthesis, with reports indicating up to a 2230-fold increase in fungal cultures (Giri and Zaheer 2016). Beyond enhancing the biosynthesis of previously characterized compounds, elicitors can activate new metabolic pathways. Alghuthaymi et al. (Alghuthaymi et al. 2022) demonstrated that MeJA treatment significantly increased rosmarinic acid accumulation (117 mg/g dry weight) in *Halodule pinifolia* cultures, along with the synthesis of 47 additional metabolites, including flavonoids and phenolic acids. These findings suggest that elicitors modulate metabolic production and trigger the synthesis of previously undetected compounds, expanding the repertoire of metabolites with potential biotechnological applications. The chemical detection of cyclic peptides [e.g., maculosin, cyclo(-Pro-Val)] in the fungal extracts via LC–MS/MS confirms the activity of this specific biosynthetic machinery. The presence of these types of compounds, which are known to mediate ecological interactions and exhibit notable bioactive properties in other fungi, suggests a potential role in defense mechanisms or ecological adaptation for this strain. For example, griseaketides produced by *Penicillium* spp. have shown antibacterial and antifungal activity, indicating a defensive role in their natural environment (Valente et al. 2020). Co-culture experiments and stress exposure have been shown to induce the production of antimicrobial cyclic peptides in phytopathogenic fungi (Soledade and Chumala 2006), while in endophytic fungi, these metabolites have been linked to microbial competition within plant tissues (Zhou et al. 2011). The detection of the betaine lipid DGTS (18:2) suggests a potential role in membrane remodeling. This finding is intriguing given that in other organisms like *Chlorella kessleri*, specific DGTS species are known to replace phosphatidylcholine under phosphorus-deficient conditions as part of a strategy to maintain membrane integrity. While the detection of DGTS (18:2) in our system is consistent with a possible adaptive response to stress, a direct causal relationship in this specific context remains to be established through future targeted lipidomic studies under controlled conditions (Riekhof et al. 2014). Similar findings have been reported in fungi, where phosphate starvation triggers the replacement of phospholipids with DGTS, highlighting its conserved role in stress adaptation. Furthermore, the detection of glycosylated flavonoids, such as 7-O-methyleriodictyol 3’’-O-glucoside, is of interest due to the documented pharmacological properties of this class of compounds. For instance, studies in other systems have shown that glycosylation can enhance flavonoid solubility and stability (Krawczyk‐łebek et al. 2022), which may improve their antioxidant capacity. This is supported by work such as that of Liu et al. (Liu et al. 2022), where the induction of glycosylated flavonoids under stress was correlated with reduced oxidative damage. Thus, the presence of these metabolites in our system warrants further investigation to assess their actual antioxidant and pharmacological potential (Behr et al. 2020).

The integration of genomic and metabolomic data is essential for understanding the biosynthetic potential of an organism. Three cyclic peptides, cyclo(Pro-Val), cyclo(L-Phe-D-Pro), and cyclo(L-Pro-L-Leu), were detected through LC–QTOF–MS/MS metabolomic analysis, annotated via GNPS, and classified as amino acid and peptide derivatives by CANOPUS (Toghueo et al. 2020). These compounds were primarily induced under LPS treatment and expressed in culture media such as YES, IMA, and ISP-4. Additionally, genomic analysis using antiSMASH and BGC clustering via BiG-SCAPE revealed NRPS-type clusters with high similarity to known BGCs from the MIBiG database, supporting the hypothesis that these metabolites originate from biosynthetic gene clusters within *P. australis*. However, despite the use of elicitors such as NO and LPS, metabolites for some predicted BGCs were not detected. This could indicate a requirement for more specific activation signals, or it could suggest that novel metabolites were produced but remained among the unannotated features in our metabolomic datasets, highlighting the potential for discovering new chemistry (Pfannenstiel and Keller 2019). Additionally, certain clusters may be epigenetically regulated, produce metabolites at very low concentrations, or generate unstable or non-ionizable compounds under the applied analytical conditions (Robey et al. 2020; Xue et al. 2023). Tools such as GNPS and SIRIUS provide predictive metabolomic analyses, aiding in metabolite annotation and structural classification. Nevertheless, these platforms rely on mass spectrometry-based predictions; thus, further validation is required using techniques capable of elucidating the precise chemical structure, such as NMR or X-ray crystallography. These findings highlight the need to combine chemical elicitation with transcriptomic strategies and structural analytical methods to fully access the organism’s metabolic repertoire (Clevenger et al. 2017).

These observations highlight the necessity of integrated approaches that link genetic pathways to the production of novel compounds. Exploiting the potential of microorganisms to uncover new molecules could be vital in addressing the global demand for developing safer, more cost-effective, and efficient drugs for various diseases. The present moment offers an ideal opportunity to reinvigorate and intensify our dedication to microorganism-inspired drug discovery.

## Conclusion

This study reports the first annotated genome of *P. australis* UA-032-E, revealing its considerable biosynthetic potential through the identification of 44 BGCs using antiSMASH, including NRPS, type I PKS, and hybrid systems. Analysis with BiG-SCAPE grouped these BGCs into 41 families, most of which were singletons, suggesting low redundancy and high structural diversity. The application of elicitors such as SNP and LPS in different culture media enabled the activation of previously undetected metabolic pathways. Metabolomic analysis via HPLC–QTOF–MS/MS, complemented with GNPS and SIRIUS, allowed the detection of 75 features. Among them, compounds such as cyclodipeptides [cyclo-(Pro-Val), cyclo-(Leu-Leu)], maculosin, and betaine lipids like DGTS 18:2 were identified, which have been associated in the literature with potential roles in stress adaptation or biological activities. The YES + LPS condition showed the highest metabolite diversity, indicating that this combination of medium and elicitor may be favorable for the expression of specialized metabolism. Together, these results highlight the usefulness of integrating genomic and metabolomic approaches to explore the chemical potential of psychrophilic fungi. The genomic characterization presented here constitutes a fundamental resource for future functional studies and bioprospecting efforts aimed at elucidating biosynthetic pathways and characterizing metabolites with potential biotechnological applications.

## Additional information

### Use of AI

We used AI-powered tools, including Paperpal, solely for the purpose of improving the language, clarity, and readability of the manuscript.

### Adherence to national and international regulations

All the fungal strains used in this study have been legally obtained, respecting the Convention on Biological Diversity (Rio Convention).

### Author ORCIDs

Karla Leal https://orcid.org/0000-0001-7526-464X

Juan Machuca https://orcid.org/0009-0007-5618-0302

María José Contreras https://orcid.org/0000-0002-1346-9479

Leticia Barrientos https://orcid.org/0000-0002-5344-054X

Kattia Nuñez-Montero https://orcid.org/0000-0002-8629-5107

Estefanía Chavarría https://orcid.org/0009-0009-2234-465X

Pablo Bruna https://orcid.org/0009-0005-6327-1785

Isabel Iturrieta-González https://orcid.org/0000-0001-8426-9530

### Data availability

All of the data that support the findings of this study are available in the main text or Supplementary Information.

## Supplementary materials

Supplementary material 1 Phylogenetic classification of isolate UA-032-E within the *P. australis* clade and classification and network analysis of BGC families Data type docx Explanation note Suppl. material 1 provides additional data supporting this study. Section S1 presents the phylogenetic placement of *Pseudogymnoascus australis* UA-032-E using multilocus sequence analysis. Section S2 details the classification and network analysis of BGCs identified in the genome. Together, these data highlight the strain’s evolutionary relationships and biosynthetic potential. https://binary.pensoft.net/file/1537896This dataset is made available under the Open Database License (http://opendatacommons.org/licenses/odbl/1.0/). The Open Database License (ODbL) is a license agreement intended to allow users to freely share, modify, and use this Dataset while maintaining this same freedom for others, provided that the original source and author(s) are credited. Karla Leal, Juan Machuca, David Madariaga, María José Contreras, Leticia Barrientos, Kattia Nuñez-Montero, Estefanía Chavarría, Pablo Bruna, Isabel Iturrieta-González

## References

[B1] Albarano L, Esposito R, Ruocco N et al. (2020) Genome mining as new challenge in natural products discovery. Marine Drugs 18: 199. 10.3390/md18040199 PMC7230286 32283638

[B2] Alghuthaymi MA, Danaraj J, Albarakaty FM et al. (2022) Elicitor-Induced Metabolomics Analysis of Halodule pinifolia Suspension Culture for an Alternative Antifungal Screening Approach against Candida albicans. Journal of Fungi 8: 609. 10.3390/jof8060609 PMC9224785 35736092

[B3] Allen BM, Drott M, Nickles GR et al. (2024) Variation in Biosynthetic Gene Clusters Among Lifestyles Across Kingdom Fungi. bioRxiv. 10.1101/2024.09.14.613087

[B4] Alves BVB, Borges LJ, Hanna SA et al. (2025) Pigment production by *Pseudofusicoccum* sp.: Extract production, cytotoxicity activity, and diketopiperazines identified. Microorganisms 13: 277. 10.3390/microorganisms13020277 PMC11857561 40005644

[B5] Anon (2025) Anon GitHub - s-andrews/FastQC: A quality control analysis tool for high throughput sequencing data. [Available from:] https://github.com/s-andrews/FastQC [February 27, 2025]

[B6] Antipova TV, Zaitsev KV, Zhelifonova VP et al. (2023) The potential of arctic *Pseudogymnoascus* fungi in the biosynthesis of natural products. Fermentation 9: 702. 10.3390/fermentation9080702

[B7] Aylward J, Wilson AM, Visagie CM et al. (2024) IMA Genome – F19. IMA Fungus 15: 12. 10.1186/s43008-024-00142-z PMC11149380 38831329

[B8] Bach TNQ, Cao DT, Hoang THL et al. (2022) Cyclodipeptides isolated from a marine-derived fungus *Penicillium chrysogenum* M612 of Bai Tu Long Sea, Quang Ninh, Vietnam. IFMBE Proceedings 85: 527–537. 10.1007/978-3-030-75506-5_45

[B9] Bateman A, Martin MJ, Orchard S et al. (2023) UniProt: the Universal Protein knowledgebase in 2023. Nucleic Acids Research 51: D523–D531. 10.1093/nar/gkac1052 PMC9825514 36408920

[B10] Behr M, Neutelings G, El Jaziri M et al. (2020) You want it sweeter: How glycosylation affects plant response to oxidative stress. Frontiers in Plant Science 11: 571399. 10.3389/fpls.2020.571399 PMC7525049 33042189

[B11] Benaud N, Edwards RJ, Amos TG et al. (2021) Antarctic desert soil bacteria exhibit high novel natural product potential, evaluated through long-read genome sequencing and comparative genomics. Environmental Microbiology 23: 3646–3664. 10.1111/1462-2920.15300 33140504

[B12] Bergmann S, Schümann J, Scherlach K et al. (2007) Genomics-driven discovery of PKS-NRPS hybrid metabolites from *Aspergillus nidulans*. Nature Chemical Biology 3: 213–217. 10.1038/nchembio869 17369821

[B13] Bhaskar R, Xavier LSE, Udayakumaran G et al. (2021) Biotic elicitors: a boon for the *in-vitro* production of plant secondary metabolites. Plant Cell, Tissue and Organ Culture (PCTOC) 149: 7–24. 10.1007/s11240-021-02131-1

[B14] Blin K, Shaw S, Kloosterman AM et al. (2021) antiSMASH 6.0: improving cluster detection and comparison capabilities. Nucleic Acids Research 49: W29–W35. 10.1093/nar/gkab335 PMC8262755 33978755

[B15] Blin K, Shaw S, Vader L et al. (2025) antiSMASH 8.0: extended gene cluster detection capabilities and analyses of chemistry, enzymology, and regulation. Nucleic Acids Research 53: W32–W38. 10.1093/nar/gkaf334 PMC12230676 40276974

[B16] Blum M, Chang HY, Chuguransky S et al. (2021) The InterPro protein families and domains database: 20 years on. Nucleic Acids Research 49: D344–D354. 10.1093/nar/gkaa977 PMC7778928 33156333

[B17] Cánovas D, Marcos JF, Marcos AT et al. (2016) Nitric oxide in fungi: is there NO light at the end of the tunnel? Current Genetics 62: 513–518. 10.1007/s00294-016-0574-6 PMC4929157 26886232

[B18] Cantalapiedra CP, Hernández-Plaza A, Letunic I et al. (2021) eggNOG-mapper v2: Functional annotation, orthology assignments, and domain prediction at the metagenomic scale. Molecular Biology and Evolution 38: 5825–5829. 10.1093/molbev/msab293 PMC8662613 34597405

[B19] Castaldi S, Cimmino A, Masi M et al. (2022) Bacterial Lipodepsipeptides and some of their derivatives and cyclic dipeptides as potential agents for biocontrol of pathogenic bacteria and fungi of agrarian plants. Journal of Agricultural and Food Chemistry 70: 4591–4598. 10.1021/acs.jafc.1c08139 PMC9026286 35395154

[B20] Chen S, Zhou Y, Chen Y et al. (2018) fastp: an ultra-fast all-in-one FASTQ preprocessor. Bioinformatics (Oxford, England) 34: i884–i890. 10.1093/bioinformatics/bty560 PMC6129281 30423086

[B21] Childress MK, Dragone NB, Young BD et al. (2025) Three new *Pseudogymnoascus* species (*Pseudeurotiaceae*, *Thelebolales*) described from Antarctic soils. IMA Fungus 16: e142219. 10.3897/imafungus.16.e142219 PMC11953729 40162003

[B22] Clevenger KD, Bok JW, Ye R et al. (2017) A scalable platform to identify fungal secondary metabolites and their gene clusters. Nature Chemical Biology 13: 895–901. 10.1038/nchembio.2408 PMC5577364 28604695

[B23] Collemare J, Billard A, Böhnert HU et al. (2008) Biosynthesis of secondary metabolites in the rice blast fungus *Magnaporthe grisea*: the role of hybrid PKS-NRPS in pathogenicity. Mycological Research 112(Issue 2): 207–215. 10.1016/j.mycres.2007.08.003 18272356

[B24] De Coster W, D’Hert S, Schultz DT et al. (2018) NanoPack: visualizing and processing long-read sequencing data. Bioinformatics (Oxford, England) 34: 2666–2669. 10.1093/bioinformatics/bty149 PMC6061794 29547981

[B25] Delcher AL, Bratke KA, Powers EC et al. (2007) Identifying bacterial genes and endosymbiont DNA with Glimmer. Bioinformatics 23: 673–679. 10.1093/bioinformatics/btm009 PMC2387122 17237039

[B26] Dewapriya P, Khalil ZG, Prasad P et al. (2018) Talaropeptides A-D: Structure and biosynthesis of extensively N-methylated linear peptides from an Australian marine tunicate-derived *Talaromyces* sp. Frontiers in Chemistry 6: 411960. 10.3389/fchem.2018.00394 PMC6131563 30234104

[B27] Dührkop K, Fleischauer M, Ludwig M et al. (2019) SIRIUS 4: a rapid tool for turning tandem mass spectra into metabolite structure information. Nature Methods 16: 299–302. 10.1038/s41592-019-0344-8 30886413

[B28] Flynn JM, Hubley R, Goubert C et al. (2020) RepeatModeler2 for automated genomic discovery of transposable element families. Proceedings of the National Academy of Sciences of the United States of America 117: 9451–9457. 10.1073/pnas.1921046117 PMC7196820 32300014

[B29] Ge HM, Song YC, Chen JR et al. (2006) Paranolin: a New Xanthene-Based Metabolite from *Paraphaeosphaeria nolinae*. Helvetica Chimica Acta 89: 502–506. 10.1002/hlca.200690051

[B30] Giri CC, Zaheer M (2016) Chemical elicitors versus secondary metabolite production in vitro using plant cell, tissue and organ cultures: recent trends and a sky eye view appraisal. Plant Cell, Tissue and Organ Culture (PCTOC) 126: 1–18. 10.1007/s11240-016-0985-6

[B31] Gurevich A, Saveliev V, Vyahhi N et al. (2013) QUAST: quality assessment tool for genome assemblies. Bioinformatics 29: 1072–1075. 10.1093/bioinformatics/btt086 PMC3624806 23422339

[B32] Hassan N, Rafiq M, Hayat M et al. (2016) Psychrophilic and psychrotrophic fungi: a comprehensive review. Reviews in Environmental Science and Bio/Technology 15: 147–172. 10.1007/s11157-016-9395-9

[B33] Hinsch J, Vrabka J, Oeser B et al. (2015) *De novo* biosynthesis of cytokinins in the biotrophic fungus *Claviceps purpurea*. Environmental Microbiology 17: 2935–2951. 10.1111/1462-2920.12838 25753486

[B34] Hoshino S, Onaka H, Abe I (2019) Activation of silent biosynthetic pathways and discovery of novel secondary metabolites in actinomycetes by co-culture with mycolic acid-containing bacteria. Journal of Industrial Microbiology & Biotechnology 46: 363–374. 10.1007/s10295-018-2100-y 30488365

[B35] Jones P, Binns D, Chang HY et al. (2014) InterProScan 5: genome-scale protein function classification. Bioinformatics (Oxford, England) 30: 1236–1240. 10.1093/bioinformatics/btu031 PMC3998142 24451626

[B36] Käll L, Krogh A, Sonnhammer ELL (2007) Advantages of combined transmembrane topology and signal peptide prediction—the Phobius web server. Nucleic Acids Research 35: W429–W432. 10.1093/nar/gkm256 PMC1933244 17483518

[B37] Khalil ZG, Kalansuriya P, Capon RJ (2014) Lipopolysaccharide (LPS) stimulation of fungal secondary metabolism. Mycology 5: 168–178. 10.1080/21501203.2014.930530 PMC4205919 25379339

[B38] Kim HW, Wang M, Leber CA et al. (2021) NPClassifier: A deep neural network-based structural classification tool for natural products. Journal of Natural Products 84: 2795–2807. 10.1021/acs.jnatprod.1c00399 PMC8631337 34662515

[B39] Korf I (2004) Gene finding in novel genomes. BMC Bioinformatics 5: 59. 10.1186/1471-2105-5-59 PMC421630 15144565

[B40] Krawczyk‐łebek A, Dymarska M, Janeczko T et al. (2022) Glycosylation of methylflavonoids in the cultures of entomopathogenic filamentous fungi as a tool for obtaining new biologically active compounds. International Journal of Molecular Sciences 23: 5558. 10.3390/ijms23105558 PMC9146141 35628367

[B41] Liu H, Chen S, Liu W et al. (2016) Polyketides with immunosuppressive activities from mangrove endophytic fungus *Penicillium* sp. ZJ-SY2. Marine Drugs 14: 217. 10.3390/md14120217 PMC5192454 27897975

[B42] Liu XQ, Cheng S, Aroca R et al. (2022) Arbuscular mycorrhizal fungi induce flavonoid synthesis for mitigating oxidative damage of trifoliate orange under water stress. Environmental and Experimental Botany 204: 105089. 10.1016/j.envexpbot.2022.105089

[B43] Lowe TM, Eddy SR (1997) tRNAscan-SE: a program for improved detection of transfer RNA genes in genomic sequence. Nucleic Acids Research 25: 955–964. 10.1093/nar/25.5.955 PMC146525 9023104

[B44] Ma H, Wang F, Jin X et al. (2021) A new diketopiperazine from an endophytic fungus *Aspergillus aculeatus* F027. Natural Product Research 35: 2370–2375. 10.1080/14786419.2019.1677652 31617784

[B45] Maansson M, Vynne NG, Klitgaard A et al. (2016) An integrated metabolomic and genomic mining workflow to uncover the biosynthetic potential of bacteria. mSystems 1. 10.1128/mSystems.00028-15 PMC5069768 27822535

[B46] Minami A, Ugai T, Ozaki T et al. (2020) Predicting the chemical space of fungal polyketides by phylogeny-based bioinformatics analysis of polyketide synthase-nonribosomal peptide synthetase and its modification enzymes. Scientific Reports 10: 13556. 10.1038/s41598-020-70177-w PMC7421883 32782278

[B47] Naquin D, d’Aubenton-Carafa Y, Thermes C et al. (2014) CIRCUS: A package for Circos display of structural genome variations from paired-end and mate-pair sequencing data. BMC Bioinformatics 15: 1–6. 10.1186/1471-2105-15-198 PMC4071023 24938393

[B48] Navarro-Muñoz JC, Selem-Mojica N, Mullowney MW et al. (2019) A computational framework to explore large-scale biosynthetic diversity. Nature Chemical Biology 16: 60. 10.1038/s41589-019-0400-9 PMC6917865 31768033

[B49] Núñez-Montero K, Quezada-Solís D, Khalil ZG et al. (2020) Genomic and metabolomic analysis of antarctic bacteria revealed culture and elicitation conditions for the production of antimicrobial compounds. Biomolecules 10: 673. 10.3390/biom10050673 PMC7277857 32349314

[B50] Ochi K, Hosaka T (2013) New strategies for drug discovery: Activation of silent or weakly expressed microbial gene clusters. Applied Microbiology and Biotechnology 97: 87–98. 10.1007/s00253-012-4551-9 PMC3536979 23143535

[B51] Ogaki MB, Teixeira DR, Vieira R et al. (2020) Diversity and bioprospecting of cultivable fungal assemblages in sediments of lakes in the Antarctic Peninsula. Fungal Biology 124: 601–611. 10.1016/j.funbio.2020.02.015 32448451

[B52] Palazzotto E, Weber T (2018) Omics and multi-omics approaches to study the biosynthesis of secondary metabolites in microorganisms. Current Opinion in Microbiology 45: 109–116. 10.1016/j.mib.2018.03.004 29656009

[B53] Pannkuk EL, Blair HB, Fischer AE et al. (2014) Triacylglyceride composition and fatty acyl saturation profile of a psychrophilic and psychrotolerant fungal species grown at different temperatures. Fungal Biology 118: 792–799. 10.1016/j.funbio.2014.06.005 25209638

[B54] Pereira H, Silva PC, Johansson B (2023) Bacteria and yeast colony PCR. Methods in Molecular Biology 2967: 209–221. 10.1007/978-1-0716-3358-8_17 37608114

[B55] Pfannenstiel BT, Keller NP (2019) On top of biosynthetic gene clusters: How epigenetic machinery influences secondary metabolism in fungi. Biotechnology Advances 37: 107345. 10.1016/j.biotechadv.2019.02.001 PMC6685777 30738111

[B56] Rawlings ND, Waller M, Barrett AJ et al. (2014) MEROPS: the database of proteolytic enzymes, their substrates and inhibitors. Nucleic Acids Research 42: D503–D509. 10.1093/nar/gkt953 PMC3964991 24157837

[B57] Riekhof WR, Naik S, Bertrand H et al. (2014) Phosphate starvation in fungi induces the replacement of phosphatidylcholine with the phosphorus-free betaine lipid Diacylglyceryl-N,N,N-Trimethylhomoserine. Eukaryotic Cell 13: 749–757. 10.1128/EC.00004-14 PMC4054272 24728191

[B58] Robey MT, Caesar LK, Drott MT et al. (2020) An interpreted atlas of biosynthetic gene clusters from 1,000 fungal genomes. Proceedings of the National Academy of Sciences 118: e2020230118. 10.1101/2020.09.21.307157 PMC8126772 33941694

[B59] Satriawan H, Teoh TC, Rizman-Idid M et al. (2024) Polar fungi *Pseudogymnoascus*: Secondary metabolites and ecological significance. Chiang Mai Journal of Science 51: e2024043. 10.12982/CMJS.2024.043

[B60] Scherlach K, Hertweck C (2009) Triggering cryptic natural product biosynthesis in microorganisms. Organic & Biomolecular Chemistry 9: 1753–1760. 10.1039/b821578b 19590766

[B61] Schümann J, Hertweck C (2007) Molecular basis of cytochalasan biosynthesis in fungi: Gene cluster analysis and evidence for the involvement of a PKS-NRPS hybrid synthase by RNA silencing. Journal of the American Chemical Society 129: 9564–9565. 10.1021/ja072884t 17636916

[B62] Shao C, Wang C, Wei M et al. (2008) Structure elucidation of two new xanthone derivatives from the marine fungus *Penicillium* sp. (ZZF 32#) from the South China Sea. Magnetic Resonance in Chemistry 46: 1066–1069. 10.1002/mrc.2293 18759333

[B63] Shi T, Yu YY, Dai JJ et al. (2021) New Polyketides from the Antarctic Fungus *Pseudo­gymnoascus* sp. HSX2#-11. Marine Drugs 19: 168. 10.3390/md19030168 PMC8004129 33809861

[B64] Shi Y, Ji M, Dong J et al. (2024) New bioactive secondary metabolites from fungi: 2023. Mycology 15: 283–321. 10.1080/21501203.2024.2354302 PMC11376311 39247896

[B65] Soledade PPC, Chumala PB (2006) Peptides and Depsipeptides from Plant Pathogenic Fungi. In: Kastin AJ (Ed.) Handbook of Biologically Active Peptides, 151–156. 10.1016/B978-012369442-3/50026-X

[B66] Stanke M, Keller O, Gunduz I et al. (2006) AUGUSTUS: ab initio prediction of alternative transcripts. Nucleic Acids Research 34: W435–W439. 10.1093/nar/gkl200 PMC1538822 16845043

[B67] Teoh TC, Rizman-Idid M, Wong HJ et al. (2024) Transcriptomic evidence of base and nucleotide excision repair mechanisms in response to UV-B radiation in an Arctic fungus *Pseudogymnoascus australis* strain HNDR4. Chiang Mai Journal of Science 51: e2024029. 10.12982/CMJS.2024.029

[B68] Ter-Hovhannisyan V, Lomsadze A, Chernoff YO et al. (2008) Gene prediction in novel fungal genomes using an ab initio algorithm with unsupervised training. Genome Research 18: 1979–1990. 10.1101/gr.081612.108 PMC2593577 18757608

[B69] Toghueo RMK, Sahal D, Boyom FF (2020) Recent advances in inducing endophytic fungal specialized metabolites using small molecule elicitors including epigenetic modifiers. Phytochemistry 174: 112338. 10.1016/j.phytochem.2020.112338 32179305

[B70] Vaishnav P, Demain AL (2011) Unexpected applications of secondary metabolites. Biotechnology Advances 29: 223–229. 10.1016/j.biotechadv.2010.11.006 21130862

[B71] Valente S, Cometto A, Piombo E et al. (2020) Elaborated regulation of griseofulvin biosynthesis in *Penicillium griseofulvum* and its role on conidiation and virulence. International Journal of Food Microbiology 328: 108687. 10.1016/j.ijfoodmicro.2020.108687 32474227

[B72] Villanueva P, Vásquez G, Gil-Durán C et al. (2021) Description of the first four species of the genus *Pseudogymnoascus* from Antarctica. Frontiers in Microbiology 12: 713189. 10.3389/fmicb.2021.713189 PMC8640180 34867840

[B73] Wang JF, Zhou LM, Chen ST et al. (2018) New chlorinated diphenyl ethers and xanthones from a deep-sea-derived fungus *Penicillium chrysogenum* SCSIO 41001. Fitoterapia 125: 49–54. 10.1016/j.fitote.2017.12.012 29269234

[B74] Wang M, Jiang X, Wu W et al. (2015) Psychrophilic fungi from the world’s roof. Persoonia: Molecular Phylogeny and Evolution of Fungi 34: 100–112. 10.3767/003158515X685878 PMC4510274 26240448

[B75] Wang M, Carver JJ, Phelan VV et al. (2016) Sharing and community curation of mass spectrometry data with Global Natural Products Social Molecular Networking. Nature Biotechnology 34: 828–837. 10.1038/nbt.3597 PMC5321674 27504778

[B76] Xu F, Wu Y, Zhang C et al. (2019) A genetics-free method for high-throughput discovery of cryptic microbial metabolites. Nature Chemical Biology 15: 161–168. 10.1038/s41589-018-0193-2 PMC6339573 30617293

[B77] Xue M, Hou X, Fu J et al. (2023) Recent advances in search of bioactive secondary metabolites from fungi triggered by chemical epigenetic modifiers. Journal of Fungi 9: 172. 10.3390/jof9020172 PMC9961798 36836287

[B78] Yang YH, Yang DS, Lei HM et al. (2020) Griseaketides A–D, new aromatic polyketides from the pathogenic fungus *Magnaporthe grisea*. Molecules 25: 72. 10.3390/molecules25010072 PMC6982942 31878244

[B79] Yin Y, Mao X, Yang J et al. (2012) dbCAN: a web resource for automated carbohydrate-active enzyme annotation. Nucleic Acids Research 40: W445–W451. 10.1093/nar/gks479 PMC3394287 22645317

[B80] Yoshinaga TT, Giovanella P, de Farias GS et al. (2024) Fungi from Antarctic marine sediment: characterization and assessment for textile dye decolorization and detoxification. Brazilian Journal of Microbiology [publication of the Brazilian Society for Microbiology] 55: 3437–3448. 10.1007/s42770-024-01485-w PMC11711567 39259479

[B81] Zhao Y, Lim J, Xu J et al. (2020) Nitric oxide as a developmental and metabolic signal in filamentous fungi. Molecular Microbiology 113: 872–882. 10.1111/mmi.14465 31968137

[B82] Zhou K, Zhang X, Zhang F et al. (2011) Phylogenetically diverse cultivable fungal community and Polyketide Synthase (PKS), Non-Ribosomal Peptide Synthase (NRPS) genes associated with the South China Sea sponges. Microbial Ecology 62: 644–654. 10.1007/s00248-011-9859-y 21519913

[B83] Zimin AV, Salzberg SL (2020) The genome polishing tool POLCA makes fast and accurate corrections in genome assemblies. PLOS Computational Biology 16: e1007981. 10.1371/journal.pcbi.1007981 PMC7347232 32589667

[B84] Zimin AV, Salzberg SL (2022) The SAMBA tool uses long reads to improve the contiguity of genome assemblies. PLOS Computational Biology 18: e1009860. 10.1371/journal.pcbi.1009860 PMC8849508 35120119

[B85] Zucconi L, Canini F, Temporiti ME et al. (2020) Extracellular enzymes and bioactive compounds from Antarctic terrestrial fungi for bioprospecting. International Journal of Environmental Research and Public Health 17: 6459. 10.3390/ijerph17186459 PMC7558612 32899827

