# Supplementary material for: Exploration of inactive metabolic pathways in Antarctic Pseudogymnoascus australis through elicitation: a genomic and metabolomic approach to investigate its biotechnological potential
Source: IMA Fungus. 2026 Feb 11;17:e156018. doi: 10.3897/imafungus.17.156018 (PMC12917493; doi:10.3897/imafungus.17.156018)
Supplement: Supplementary material 1 — Phylogenetic classification of isolate UA-032-E within the P. australis clade and classification and network analysis of BGC families [file imafungus-17-e156018-s001.docx]

**S1. Phylogenetic classification of isolate UA-032-E within the *P. australis* clade.**

The taxonomic classification of isolate UA-032-E within the *Pseudogymnoascus* genus was determined through phylogenetic analysis using combined ITS, RPB2, and Tef1 sequences. Alignments were conducted using ClustalW and MUSCLE algorithms, manually refined, and assessed using Maximum Likelihood (ML) and Bayesian Inference (BI) methods. The resulting phylogenetic tree (Figure S1) demonstrates that strain UA-032-E groups with the *P. australis* clade, including the type of strain *P. australis* CHFC-EA 567T and related strains. This grouping is corroborated by high ML bootstrap values (bs = 99%), suggesting a strong phylogenetic link to this species (Table S1). The tree also exhibits distinct separation between *P. australis* and other *Pseudogymnoascus* species, such as *P. griseus*, *P. papyriferae*, and *P. shaanxiensis*, which form well-supported clades (bs ≥ 70%). The outgroup species, *Geomyces auratus* and *Geomyces obovatus*, are positioned outside the *Pseudogymnoascus* cluster, affirming their phylogenetic distinction. These findings validate the classification of isolate UA-032-E as *P. australis* and underscore the effectiveness of phylogenetic analysis using multiple molecular markers. This integrated approach enabled the resolution of evolutionary relationships within the genus and established clear phylogenetic connections among the studied species.


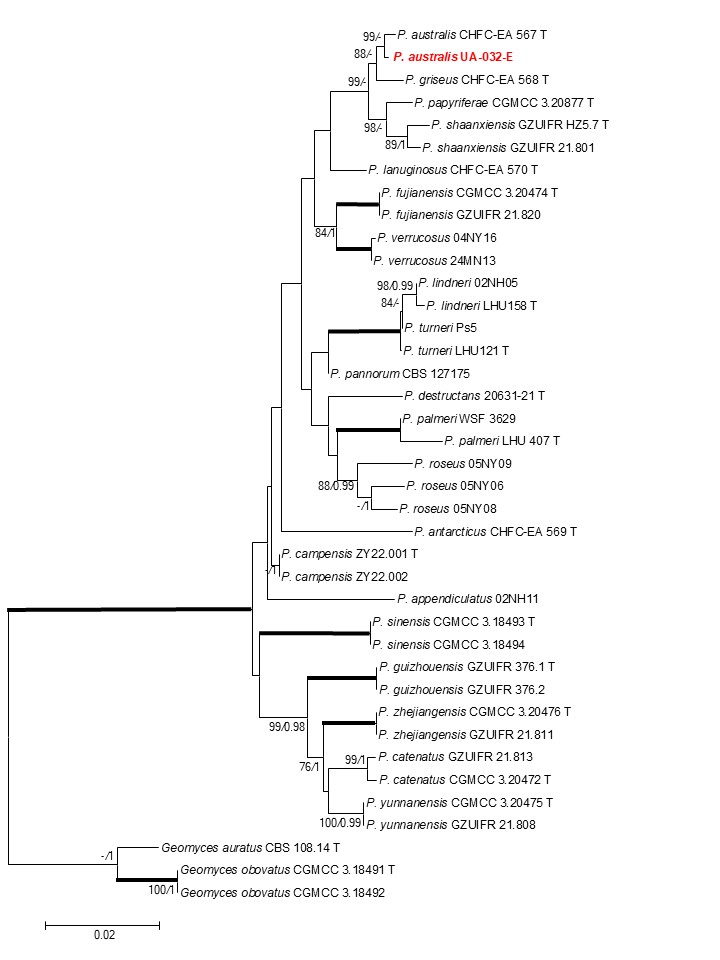


**Figure S1.** Phylogenetic tree of *Pseudogymnoascus* species based on maximum likelihood and Bayesian inference analysis, using the combined analysis of ITS, RPB2 and tef1 and rooted with *Geomyces auratus* (CBS 108.14) and *Geomyces obovatus* (CGMCC 3.18491 and CGMCC 3.18492). Bootstrap values (bs) greater than 70% and Bayesian posterior probabilities (pp) greater than 0.95 are given at the nodes (bs/pp). Bold branches indicate bs/pp of 100/1. The isolate of the present study is highlighted in bold, and red. Ex-type isolates are marked with a T.

**Table S1:** *Pseudogymnoascus* species included in the phylogenetic study and their respective GenBank accession number.

| **Species** | **Strains** | **GenBank accesion numbers** | | |
| --- | --- | --- | --- | --- |
|  |  | **ITS** | **RPB2** | **Tef1** |
| *Geomyces auratus* | CBS 108.14 (T) | KF039895 | KF017746 | KF017805 |
| *Geomyces obovatus* | CGMCC 3.18491 (T) | MT509362 | MT534216 | MT534227 |
|  | CGMCC 3.18492 | MT509363 | MT534217 | MT534228 |
| *Pseudogymnoascus antarcticus* | CHFC-EA 569 (T) | JX845280 | MN418135 | MN418131 |
| *Pseudogymnoascus appendiculatus* | 02NH11 | JX270356 | KF017704 | KF017759 |
| *Pseudogymnoascus australis* | CHFC-EA 567 (T) | MN417287 | MN418137 | MN418133 |
|  | **UA-032-E** | **PV350061** | **PV369472** | **PV369473** |
| *Pseudogymnoascus campensis* | ZY22.001 (T) | OP796795 | - | OP781429 |
|  | ZY22.002 | OP796796 | - | OP781430 |
| *Pseudogymnoascus catenatus* | GZUIFR 21.813 | MZ444078 | MZ488543 | MZ488520 |
|  | CGMCC 3.20472 (T) | MZ444080 | MZ488545 | MZ488522 |
| *Pseudogymnoascus destructans* | 20631-21 T | EU884921 | KF017747 | KF017806 |
| *Pseudogymnoascus fujianensis* | CGMCC 3.20474 (T) | MZ444084 | MZ488549 | MZ488526 |
|  | GZUIFR 21.820 | MZ444085 | MZ488550 | MZ488527 |
| *Pseudogymnoascus griseus* | CHFC-EA 568 (T) | MN417288 | MN418138 | MN418134 |
| *Pseudogymnoascus guizhouensis* | GZUIFR 376.1 (T) | MT509369 | MT534223 | MT534234 |
|  | GZUIFR 376.2 | MT509370 | MT534224 | MT534235 |
| *Pseudogymnoascus pannorum* | CBS 127175 | MH864459 | - | - |
| *Pseudogymnoascus lanuginosus* | CHFC-EA 570 (T) | MN417286 | MN418136 | MN418132 |
| *Pseudogymnoascus lindneri* | 02NH05 | JX270350 | KF017703 | KF017758 |
|  | LHU158 T | MN542212 | MN541384 | MN541383 |
| *Pseudogymnoascus palmeri* | WSF 3629 | KF039897 | KF017751 | KF017811 |
|  | LHU 407 (T) | MT988150 | MW054468 | MW054467 |
| *Pseudogymnoascus papyriferae* | CGMCC_3.20877 (T) | NR_198244 | ON568973 | ON568908 |
| *Pseudogymnoascus roseus* | 05NY06 | JX270385 | KF017709 | KF017764 |
|  | 05NY08 | JX270387 | KF017710 | KF017765 |
|  | 05NY09 | JX270388 | KF017711 | KF017766 |
| *Pseudogymnoascus shaanxiensis* | GZUIFR HZ5.7 (T) | MT509366 | MT534220 | MT534231 |
|  | GZUIFR 21.801 | MZ444066 | MZ488531 | MZ488508 |
| *Pseudogymnoascus sinensis* | CGMCC 3.18493 (T) | MT509364 | MT534218 | MT534229 |
|  | CGMCC 3.18494 | MT509365 | MT534219 | MT534230 |
| *Pseudogymnoascus turneri* | LHU121 (T) | MN542213 | MN541380 | MN541379 |
|  | Ps5 | MN542214 | MN541382 | MN541381 |
| *Pseudogymnoascus verrucosus* | 04NY16 | JX270377 | KF017707 | KF017762 |
|  | 24MN13 | JX270621 | KF017743 | KF017802 |
| *Pseudogymnoascus yunnanensis* | CGMCC 3.20475 (T) | MZ444072 | MZ488537 | MZ488514 |
|  | GZUIFR_21.808 | MZ444073 | MZ488538 | MZ488515 |
| *Pseudogymnoascus zhejiangensis* | CGMCC 3.20476 (T) | MZ444075 | MZ488540 | MZ488517 |
|  | GZUIFR_21.811 | MZ444076 | MZ488541 | MZ488518 |

**S2. Classification and Network Analysis of BGC Families.**

**The BGC analysis of the**P. australis**genome was comprehensively profiled using a bioinformatic workflow. Initial prediction was performed with antiSMASH (v8.0.1) under relaxed detection parameters, which identified 43 putative BGCs of various types, including polyketide synthases (PKS), nonribosomal peptide synthetases (NRPS), terpenes, and hybrid clusters. To elucidate the relationships and family groupings among these BGCs, the resulting GenBank files were used as input for the BiG-SCAPE (v2.0.0) pipeline. A gene cluster family (GCF) similarity network was constructed in cluster mode with the following parameters: a cutoff of 0.5, Glocal alignment, and the Legacy extend strategy for distance calculation. The analysis was inclusive of all biosynthetic categories and classes, and singleton clusters were incorporated into the final network.** Two multi-member BGC families, FAM_00001 and FAM_00002, suggest minimal genetic redundancy in *P. australis*, indicating a limited biosynthetic repertoire. Notably, the BGC family FAM_00002 displays a unique genomic arrangement, with identical assemblies found on three separate contigs (e.g., contig_7.region006, contig_7.region007, and contig_19.region001). This family comprises three NRPS-type BGCs with a modular structure featuring the canonical A–T–C domain triad, which includes PF00501 (AMP-binding; adenylation domain), PF00550 (Phosphopantetheine attachment site; thiolation domain), and PF00668 (condensation domain). This configuration is characteristic of NRPS systems that facilitate substrate activation and peptide bond formation. These clusters are predicted to produce small cyclic peptides with 2 to 4 residues, incorporating various combinations of aromatic (e.g., phenylalanine, tyrosine, tryptophan) and aliphatic (e.g., valine, isoleucine) amino acids. Interestingly, two of these NRPS closely resemble BGC0000357.5 from Penicillium rubens, a known cluster encoding diketopiperazine synthases that generate cyclopeptides with the general structure cyclo-(D-aa–L-aa–D-aa–L-aa). Conversely, the BGC family FAM_00001, with identical assemblies on two distinct contigs (scaffold_17.region002 and scaffold_13.region001), encodes a highly conserved iterative T1PKS involved in synthesizing 1,3,6,8-tetrahydroxynaphthalene (THN), a crucial precursor in the DHN-melanin biosynthetic pathway. Comparative analysis using the MIBiG database reveals that FAM_00001 shares 81% sequence identity with BGC0001258.3 from Glarea lozoyensis, a known THN-producing cluster. Functional annotation of its biosynthetic genes shows a minimal yet catalytically complete PKS module, including PF00109 (β-ketoacyl synthase N-terminal domain), PF00083 (β-ketoacyl synthase C-terminal domain), PF00698 (acyltransferase), PF16073 (ACP transacylase), PF00550 (Phosphopantetheine attachment site; ACP), PF14765 (dehydratase domain), and PF00975 (Thioesterase domain). This setup supports iterative polyketide chain elongation through decarboxylative condensations of malonyl-CoA units (facilitated by the AT domain with a conserved GQGxQ motif), intermediate shuttling by the ACP, and product release by the TE domain, ultimately leading to the formation of the naphthalene scaffold through spontaneous cyclization.

**S2a.** Similar to: NRPS clusters - **BGC0000357.5** - cyclo-(D-aa–L-aa–D-aa–L-aa).

**
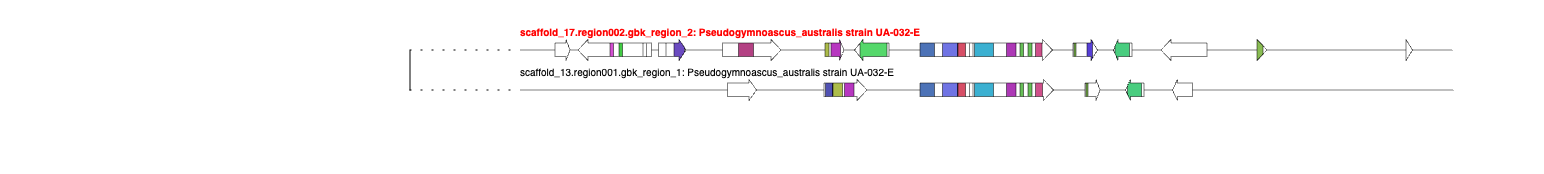
**
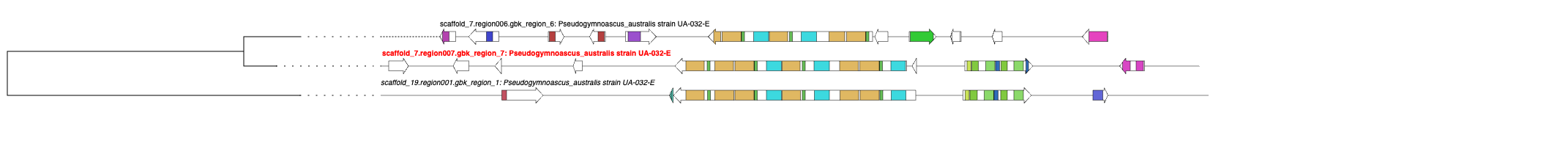


**S2b**. Similar to: T1PKS clusters - **BGC0001258.3** - 1,3,6,8-tetrahydroxynaphthalene.

**Table S2:** Functional domains and their positions in the BGCs from CC 21 and CC 53.

| **CC** | **Families** | **Pfam ID** | **Descriptions functional** |
| --- | --- | --- | --- |
| 53 | FAM_00002 | PF00668 | Condensation domain |
| 53 | FAM_00002 | PF00550 | Phosphopantetheine attachment site |
| 53 | FAM_00002 | PF00501 | AMP-binding enzyme |
| 53 | FAM_00002 | PF00668 | Condensation domain |
| 53 | FAM_00002 | PF00550 | Phosphopantetheine attachment site |
| 53 | FAM_00002 | PF00501 | AMP-binding enzyme |
| 53 | FAM_00002 | PF00668 | Condensation domain |
| 53 | FAM_00002 | PF00668 | Condensation domain |
| 53 | FAM_00002 | PF00550 | Phosphopantetheine attachment site |
| 21 | FAM_00001 | PF16073 | ACP transacylase in aflitoxin biosinthesis |
| 21 | FAM_00001 | PF00109 | β -ketoacyl synthase N-terminal domain |
| 21 | FAM_00001 | PF00083 | β -ketoacyl synthase C-terminal domain |
| 21 | FAM_00001 | PF00698 | Acyl transferase domain |
| 21 | FAM_00001 | PF14765 | Polyketide synthase dehydratase N-terminal domain |
| 21 | FAM_00001 | PF00550 | Phosphopantetheine attachment site |
| 21 | FAM_00001 | PF00550 | Phosphopantetheine attachment site |
| 21 | FAM_00001 | PF00975 | Thioesterase domain |
